# Age-related changes in error processing in young children: A school-based investigation

**DOI:** 10.1016/j.dcn.2014.02.001

**Published:** 2014-02-11

**Authors:** Jennie K. Grammer, Melisa Carrasco, William J. Gehring, Frederick J. Morrison

**Affiliations:** University of Michigan, United States

**Keywords:** Error-related negativity, Executive function, Response inhibition, School-aged children, Error positivity

## Abstract

Growth in executive functioning (EF) skills play a role children's academic success, and the transition to elementary school is an important time for the development of these abilities. Despite this, evidence concerning the development of the ERP components linked to EF, including the error-related negativity (ERN) and the error positivity (Pe), over this period is inconclusive. Data were recorded in a school setting from 3- to 7-year-old children (*N* = 96, mean age = 5 years 11 months) as they performed a Go/No-Go task. Results revealed the presence of the ERN and Pe on error relative to correct trials at all age levels. Older children showed increased response inhibition as evidenced by faster, more accurate responses. Although developmental changes in the ERN were not identified, the Pe increased with age. In addition, girls made fewer mistakes and showed elevated Pe amplitudes relative to boys. Based on a representative school-based sample, findings indicate that the ERN is present in children as young as 3, and that development can be seen in the Pe between ages 3 and 7. Results varied as a function of gender, providing insight into the range of factors associated with developmental changes in the complex relations between behavioral and electrophysiological measures of error processing.

## Highlights

• Correlates of children's response inhibition were evaluated using a Go/No-Go task.
• The ERN and Pe were present for children from 3 to 7.
• Response inhibition, as evidenced by faster, more accurate responses, increased with age.
• Age-related changes were identified in the Pe, but not in the ERN.
• Girls were more accurate and showed elevated Pe amplitudes relative to boys.

## 1 Age-related changes in error processing in young children

The transition to elementary school is characterized by rapid growth in the development of executive functioning (EF) skills, including response inhibition, working memory, and attention control. During this important developmental period, children also face increasing demands in academic domains, and there is growing evidence for the importance of EF for children's academic achievement (e.g., McClelland et al., 2007), particularly for those growing up in poverty (Raver et al., 2011). Although it is clear that early childhood is an important time for the development of EF, evidence concerning age-related behavioral and neurological changes associated with these abilities across the transition into elementary school in diverse groups of children is lacking. An increasing number of studies have contributed to the understanding of EF in childhood by examining the electrophysiological correlates of conflict-resolution and response-inhibition including the error related negativity (ERN) and the error positivity (Pe) (for a review, see Tamnes et al., 2013). Building on this work, in an effort to understand the neurological changes associated with EF across the school transition, this investigation examined the age-related changes in the ERN and Pe in preschool and elementary school-aged children.

### 1.1 The development of executive skills

Development of the group of skills identified as executive function has received increasing attention from both basic and applied researchers (Anderson, 2002, Duckworth and Seligman, 2006, Luciano, 2003, Zelazo et al., 2004). Developmental researchers have focused on the emergence and growth of executive functioning from infancy to early adulthood (Welsh, 2001). Along with educational researchers, they have sought to understand the interplay between maturational and environmental factors in shaping development of executive skills as well as in the growing concern about the role of variability in children's self-control (including gender differences) on American children's poor academic achievement, emerging even before children start school (Duckworth and Seligman, 2006, Matthews et al., 2009, McClelland et al., 2000). From a different perspective, neuroscientists have long noted that brain areas subserving basic cognitive functions such as attention and memory are distinct from those that integrate and coordinate these abilities (e.g., Luria, 1966). More recent studies have explored how these skills differ in children and adults (Welsh et al., 2006). Cognitive scientists have been analyzing the underlying components of executive functioning (attentional control/flexibility, working memory, response inhibition, planning) to ascertain their structure and function (Miyake et al., 2000, Zelazo et al., 2004).

While definitions and emphases vary, there is broad agreement that executive functions refer to cognitive skills utilized for purposeful, future-oriented behavior that allow for flexible adaptation to changing task demands, including regulation of attention, inhibition of inappropriate responses, coordination of information in working memory, and organization and planning of adaptive behavior (e.g., Blair, 2002, Eslinger, 1996, Klein, 2003, Shonkoff and Phillips, 2000, Welsh, 2002, Zelazo and Frye, 1998, Zelazo et al., 2003). In older children and adults, conceptualizations of EF include inhibitory control, working memory (updating), cognitive control (Botvinick et al., 2001), and cognitive flexibility/shifting (Monsell, 2003).

There is currently a debate regarding the composition of EF in early childhood, with many positing that the composition of EF could change with development (McAuley and White, 2010). Recently, Miyake and Friedman (2012) proposed that there are aspects of EF tasks that are shared across components (reflecting unity, or a “common EF”), but also distinct, separable, components not accounted for by common EF. Uncertainty surrounding the development of EF is confounded by differential sensitivity of behavioral measurement across age and other fundamental processes (e.g., speed of processing). Regardless of this ongoing debate, three key components commonly emphasized in the study of EF in young children encompass response inhibition, working memory, and attentional control.

In addition to growing evidence regarding the composition of EF in early childhood, there is mounting evidence that the development of executive skills occurs in context. For example, growing up in poverty negatively impacts U.S. children's EF skills, as well as the neural development thought to subserve EF abilities (e.g., Noble et al., 2007, Hackman et al., 2010). Moreover, recent research indicates that chronic poverty is particularly detrimental to children's EF (Raver et al., 2013). Although the wealth of findings point to the importance of EF skills for the academic success of children in general (McClelland et al., 2007), there is reason to suspect that these skills might be particularly important for children growing up in poverty (Raver et al., 2011).

### 1.2 Response monitoring

When considering the development of children's executive skills, studies have focused on changes in ERP components that reflect a network of structures, including the anterior cingulate cortex (ACC) and lateral prefrontal cortex, involved in detecting response conflict and attention control. The ACC has been implicated in cognitive control functions, which are thought to enable the brain to adapt behavior to changing task demands and environmental circumstances (Botvinick et al., 2001, Ridderinkhof et al., 2004). These cognitive functions include processes that detect when control is needed as well as the processes that implement control by changing the focus of attention, altering response strategies, and so on. Although there is limited longitudinal data regarding the development of the ACC, evidence from cross-sectional studies conducted in adolescence and later childhood provide some evidence for relations between developmental changes in the ACC and concurrent age-related growth observed in performance monitoring (Tamnes et al., 2013).

To this end, developmental researchers have been interested in components observed when subjects process stimuli that reflect conflicting demands of the tasks including the medial-frontal N2 potentials and frontal P3 potentials that follow them (e.g., Rueda et al., 2005, Rueda et al., 2004a, Rueda et al., 2004b). Complementing these components are those associated with the responses to stimuli in conflict tasks: the error-related negativity (ERN), a medial-frontal negativity similar to the N2, and error positivity (Pe), a positivity similar to the P3 (Arbel and Donchin, 2009, Overbeek et al., 2005).

First identified over 20 years ago (Falkenstein et al., 1991, Gehring et al., 1993), the ERN is a response-locked negative deflection usually seen at midline frontocentral scalp locations that peaks 50–100 ms following an erroneous response in speeded choice reaction time tasks. A smaller negativity, the Correct-Response Negativity (CRN), can also been identified on correct trials at the same latency as the ERN (see Gehring et al., 2012, for a review). Because commission of an error provides an indication that cognitive control is needed, theories of the ERN have tended to argue for its role in detecting the need for or in implementing cognitive control.

The presence of the ERN is often accompanied by the Pe, a positivity reaching maximum amplitude between 200 and 400 ms after the commission of an error that usually follows the ERN (Overbeek et al., 2005). Although the functional significance of the Pe is still unclear, the Pe has been associated more frequently than the ERN with conscious awareness of having made an error (Nieuwenhuis et al., 2001). Consistent with this, the Pe is thought to capture affective responses to committing an error, awareness of having made an error, or processing related to adapting response strategies after a mistake has been made (Falkenstein, 2004, Overbeek et al., 2005).

### 1.3 Developmental changes in the ERN

In contrast to the extensive literature in adult populations (for a review, see Gehring et al., 2012), much less is known about the development of the ERN. Early reports of the ERN involving children and adolescents were aimed at determining the earliest age at which the ERN could be identified. These initial studies indicated that clear ERNs were not present in children under 12 years of age, suggesting that mid-to-late adolescence was an important period for the development of this neural response (Davies et al., 2004, Ladouceur et al., 2004, Ladouceur et al., 2007). Subsequent reports indicated that ERN was observable in groups of children ranging in age from 7 to 11 (Kim et al., 2007, Wiersema et al., 2007), with non-significant increases in ERN amplitude observed with increasing age. More recent investigations, involving increasingly developmentally sensitive measures, have demonstrated that the ERN can be identified in groups of children as young as 5 and 6 years old (Torpey et al., 2009, Torpey et al., 2012). Moreover, Brooker et al. (2011) examined ERN data from a sample of children between 4 and 8 years of age. Although regression analyses failed to reveal an overall change in the ERN difference score as a function of age, girls showed a trend-level increase across that age range. However, drawing conclusions regarding the presence of the ERN in the youngest children in the sample is challenging, as published waveform data did not show comparisons of younger and older children.

Cross-sectional evidence portrays the development of the ERN as gradually occurring across childhood into adolescence (e.g., Davies et al., 2004, Ladouceur et al., 2004, Ladouceur et al., 2007, Kim et al., 2007, Santesso and Segalowitz, 2008; for a review, see Tamnes et al., 2013), with the ERN seen in young children tending to be smaller than that observed in adolescent and adult populations. However, drawing conclusions regarding the development of this component from the existing literature is challenging. The majority of reports of the development of the ERN have compared performance of relatively small groups of children and adolescents varying widely in age. Measurement confounds complicate conclusions regarding development because age related differences in the ERN have been shown to vary with the complexity of the task (Hogan et al., 2005). Although longitudinal data are necessary to fully capture developmental trajectories, a comparison of larger numbers of children across tighter windows of age would contribute to a better understanding of developmental trends in younger children.

Researchers assessing the ERN in younger populations have used a number of approaches in an attempt to characterize the component in ways that are sensitive to developmental differences. In adults, the ERN is typically described as being maximal at midline frontocentral scalp locations, and is frequently identified at FCz (Gehring et al., 2012). In contrast, in children, the ERN is often reported as being maximal somewhat more posteriorally, at Cz (e.g., Davies et al., 2004, Ladouceur et al., 2004, Torpey et al., 2012). Although researchers have adopted a range of alternative approaches for evaluating error processing in young children, such as examining a difference score in which the CRN amplitude is subtracted from the ERN (ΔERN; e.g., Torpey et al., 2012), additional questions regarding variation in the location, latency, and amplitude of the ERN in children relative to adolescents and adults remain.

### 1.4 Developmental trends in the Pe

Less is known about the Pe, and the few studies that have explored it in children indicate that the amplitude of this component is similar in children and adults (Davies et al., 2004, Wiersema et al., 2007). These reports stand in contrast to developmental data on the processes thought to be reflected by the Pe. In addition to being linked to an individual's ability to learn from his or her mistakes, the Pe is also associated with individuals with a growth mindset, or an individual's perception of the origins of one's abilities (Moser et al., 2011).

When considering associations between developmental changes in the Pe and children's executive skills, it is important to note that the Pe has been associated with post-error slowing. This slowing is often seen as an adaptive behavior reflecting learning associated with having committed an error previously (Hajcak et al., 2003). Given that the Pe is often described as reflecting the types of affective or reflective processes that develop gradually across childhood, one might anticipate that there would be observable changes in the Pe during the same periods of time that children are becoming more adept at metacognitive reflection and monitoring (e.g., Schneider, 2010). In the present investigation, we explore the development of the Pe to characterize changes in this component across an age period associated with advances in both EF and metacognition.

### 1.5 Relating behavior and ERPs in young children

There is a great deal of evidence for developmental changes in children's behavioral performance on tasks used to assess response monitoring, indicating that children become increasingly fast and accurate with age. Relations between amplitudes of the ERN and the Pe have also been identified. For example, controlling for age, Torpey and colleagues (2012) reported that children who were less accurate and faster to respond on error trials showed smaller differences between ERN and CRN amplitudes. In addition, they also found that Pe amplitudes were consistently related to children's behavioral performance as measured by accuracy and reaction time on error and correct trials.

### 1.6 Gender differences in cognitive control

Just as there is a debate regarding gender differences in EF as assessed behaviorally in young children, it is also unclear if there are differences in the ERN and Pe as a function of gender. Davies and colleagues (2004) found evidence for a pattern of gender differences in the ERN that only emerged with increases in age. Similarly, as noted above, Brooker et al. (2011) reported data consistent with an age by gender interaction in younger children. In contrast, although there is often evidence for gender differences in both reaction time and accuracy, these differences in the ERN are not observed in every sample (e.g., Santesso et al., 2006, Kim et al., 2007, Torpey et al., 2012). Moreover, there is currently no reported evidence for gender differences in the Pe (Santesso and Segalowitz, 2008, Santesso et al., 2006, Torpey et al., 2012). To date, the role of gender in early error processing is still unclear, with few studies exploring these differences in young children. However, consistent with anecdotal evidence of the regulatory skills of little girls relative to boys, an increasing number of reports suggest that girls outperform boys on behavioral tasks involving error monitoring (e.g., Matthews et al., 2009). To this end, in this investigation we examine relations between behavioral and electrophysiological measures of error monitoring and gender.

### 1.7 Considering the development of error processing in representative groups of children

Increases in collective understanding of the importance of EF skills for *all* children (e.g., Raver et al., 2013) have not been met with commensurate increases in neurophysiological data on representative groups (see e.g., Falk et al., 2013). The majority of studies involving ERP measures and young children have been conducted in laboratory settings. Although some of this work has focused on atypical development – e.g., studies of the ERN in children with obsessive compulsive disorder (e.g., Hanna et al., 2012), behavioral inhibition (McDermott et al., 2009), or anxiety (Meyer et al., 2012) – less emphasis has been placed on diversity within these samples. Indeed, relatively few reports involving child ERP data provide any information regarding demographic information of the participants (with the exception, for example, of Brooker et al., 2011, Torpey et al., 2012). As a result, the extent to which generalizations can be made regarding the development of ERP components related to executive skills is limited.

In an effort to explore the feasibility of collecting data on the ERP correlates of EF, this investigation was conducted directly in elementary schools, with the explicit goal of obtaining a representative school-based sample of children. Children participated during the school day in small conference rooms in the office or media center in their school temporarily set up for ERP recordings. In this way, obstacles to participation typically encountered in the laboratory–including those related to scheduling and transportation – were removed, allowing larger groups of children the opportunity to be a part of the investigation.

### 1.8 The current study

Given that the developmental literature provides extensive evidence for rapid growth in the development of executive skills in early childhood, one might expect to observe similar changes in children's error processing during this same period of time. Evidence suggests that developmentally appropriate measures are important for assessing children's executive skills (McDermott et al., 2007). Thus it is possible that the divergent pictures of development as seen in behavioral assessments of EF and ERP data stem from distinctions among the tasks that have been used to measure error processing in young children. As such, it seems to be particularly important to implement developmentally appropriate measures to tap into the ERN and related processes in young children.

Moreover, although research highlighting the importance of executive skills for children's academic success has focused largely on the importance of these skills for more diverse, representative groups of children (e.g., Blair and Razza, 2007, McClelland et al., 2007), the vast majority of data on the neurophysiological correlates of these skills has been collected in the laboratory with relatively homogeneous subsets of participants. The extent to which efforts are made to employ lab-based techniques outside of the laboratory in the service of recruiting and collecting ERP data on socioeconomically and ethnically representative groups of children has direct implications for the understanding of developmental patterns of executive skills.

Accordingly, this investigation was designed to explore the development of response-inhibition in 3–7 year old children in a representative school-based sample. Employing a developmentally appropriate Go/No-go task, designed specifically to be easily understood by children in preschool, yet engaging and challenging enough to elicit errors in older children, the goals of this work were to (1) evaluate the use of a child-friendly go/no-go task (McDermott et al., in preparation) to elicit the ERN and the Pe in children between the ages of 3 and 7, (2) examine age-related differences in the ERN and the Pe across the school transition, and (3) to explore gender differences in these components.

## 2 Method

### 2.1 Participants

Participants included 95 children (49 girls, 46 boys). On average children were 5.98 years old (SD = .87, range = 3.3–7.7 years). Children were recruited directly from Head Start, kindergarten, and first grade classrooms in two elementary schools. All children in participating classrooms were invited to participate in the investigation, with between 4 and 13 children in each classroom agreeing to do so. The diversity of the sample reflected the demographic characteristics of participating classrooms, and the Midwestern suburban area from which the participants were drawn, with 75% of the families describing their ethnicity as Caucasian, 12% as African American, 5% as Hispanic, 5% as Asian, and 3% as being of mixed ethnicity. Of the total sample, 81% of the children lived households where one or more of their parents had attended college, and 15% reported annual household income below $25,000.

### 2.2 Task

Participants performed a child-friendly Go/No-go task based on the task developed by McDermott and colleagues (2014). In this task, called the Zoo Game, children are told that they are playing a game in which their goal was to help a zookeeper. The children are also told that there are animals loose in the zoo, and are asked to help the zookeeper catch all of the animals so that they can be put back into their cages. Children are then informed that three of the animals, friendly orangutans, are also helping the zookeeper, so they should not be put back in their cages. In this way, children were asked to press a button as quickly as they could every time they saw an animal (Go Trials) but to inhibit their response each time they saw an orangutan (No-Go trials).

**Fig. 1:**
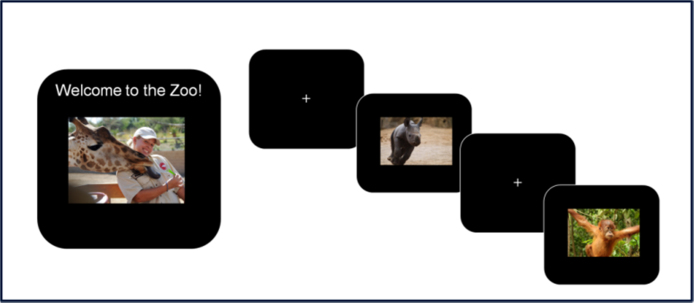
The Go/No-Go zoo task.

The first phase of the task involved a brief practice block consisting of 12 trials, 9 with zoo animals and 3 with orangutans. The children then completed 8 blocks of the task, each with 40 trials (each including 10 images of the orangutans and 30 novel zoo animal pictures), for a total of 320 trials. As is portrayed in Fig. 1, each animal image was preceded by a fixation cross displayed for a randomized interval ranging between 200 and 300 ms. The stimuli were presented for 750 ms, followed by a blank screen for 500 ms. Responses could be made while the stimulus was on the screen or at any point during the following 500 ms. Each block consisted of novel sets of animal photographs, and each set was balanced with respect to color, animal type, and size.

During the testing session, children also completed a brief working memory task whose results are not reported here.

### 2.3 Procedure

**Fig. 2:**
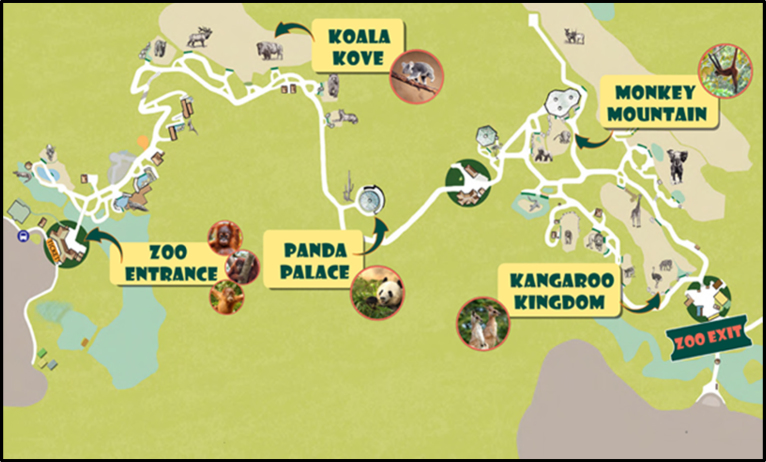
Zoo map.

All assessments were conducted in conference rooms in children's elementary schools by two experimenters. Children were excused from class for the assessment. Each participant was seated directly in front of the computer monitor and told to place equal emphasis on speed and accuracy in responding. Following a practice block of 12 trials, participants completed 8 blocks of 40 trials. Children were given performance feedback of either “Try to catch them even faster next time!” or “Watch out for the orangutan friends!” after each block of the task. The prompt children received was automatically calculated as a function of accuracy in the preceding block in an effort to yield error rates of approximately 10%, ensuring an adequate number of trials for stable error-related waveforms. To increase compliance and reduce fidgeting during ERP recording, children were given brief breaks between blocks called “Wiggle Time”. Feedback was also provided, in the form of a “Zoo Map” before the beginning of the task and after blocks 2, 4, 6, 7, and 8 (see Fig. 2) allowing children to monitor their progress through the task.

### 2.4 Electrophysiological recording, data reduction, and analysis

The EEG was recorded from DC-104 Hz with 32 Ag/AgCl scalp electrodes, two mastoid electrodes, and two vertical and two horizontal electro-oculogram electrodes, using the BioSemi ActiveTwo system. Data were recorded referenced to a ground formed from a common mode sense active electrode and driven right leg passive electrode (see http://www.biosemi.com/faq/cms&drl.htm). Data were digitized at 512 Hz and resampled offline at 256 Hz. Prior to eye movement correction, EEG data were screened using automated algorithms that rejected individual sweeps in which (a) the absolute voltage range for any individual electrode exceeded 500 μV (the range was somewhat large so that trials would not be eliminated because of EOG blinks and eye movements that could be corrected by subsequent ocular movement correction), (b) a change greater than 50 μV was measured from one datapoint to the next, or (c) the data deviated by more than +25 or −100 dB in the 20–40 Hz frequency window (for detecting muscle artifacts). Data were also screened by visual inspection. Ocular movement artifacts were then corrected using the algorithm described by Gratton et al. (1983). Waveforms shown in figures were filtered with a nine-point Chebyshev II low-pass, zero-phase-shift digital filter (Matlab R2010a; Mathworks, Natick, MA), with a half-amplitude cutoff at approximately 24 Hz.

#### 2.4.1 Behavioral Measures

Behavioral measures included the number and percentage of error and correct trials for each subject as well as children's reaction times. Children could be correct on both Go trials by correctly responding to any animal that was not an orangutan and on No-Go trials by correctly inhibiting a response when seeing an orangutan. Errors were evaluated only for No-go trials, where children committed errors of commission by responding.

Participants with fewer than 6 usable error trials overall were not included in these analyses (*n* = 7). For some children, technical errors resulted in 1 or 2 missing blocks of EEG data. Because all of these children maintained the necessary number of error trials to be evaluated in the analyses in the remaining blocks, their ERP data were retained and the percentage of the total accurate and error trials for the retained blocks are reported and used in all subsequent analyses reported below. No differences with respect to age, gender, or grade were found between children who contributed data and those with an insufficient number of errors in the sample. Average reaction times on error and correct trials were calculated separately. Trials with reaction times that occurred after 1250 ms were not included.

#### 2.4.2 ERP measures

Based on visual inspection of the grand ERPs and in accordance with previously published reports of data involving young children, the ERN, CRN, and Pe were quantified using mean amplitude measures relative to a pre-response baseline of −200 to −100 ms along the midline (FCz, Cz, and Pz). The mean amplitude of the ERN was computed on incorrect-response trials in a window from 0 to 50 ms following the response. The CRN consisted of the same measure computed on correct response trials. Consistent with previous studies, the ΔERN was also evaluated, and was calculated by subtracting the CRN from the ERN waveforms. The Pe was computed on incorrect and correct response trials in a window from 200 to 500 ms. Based on visual inspection of age-related changes in the Pe, a difference score was also calculated (ΔPe) to evaluate relative differences in the amplitude of the Pe across error and correct trials.

## 3 Results

Children's behavioral and ERP data were first evaluated on average for children with sufficient numbers of error trials (*n* = 88). Following a description of results drawn from the entire sample, age-related differences in behavior and error-related ERPs are described. Finally, gender differences in these measures are also reported. All ERP components were examined using repeated measures ANOVAs. Because of the repeated-measures structure of the data, a Greenhouse–Geisser correction was employed in all analyses where appropriate. ERN and Pe amplitudes were further compared through the use of *t*-tests, and relations between behavioral performance on the task, ERP amplitudes, and age were explored through correlational and multiple regression analyses.

### 3.1 Behavioral measures

Children's accuracy and reaction time data on error and correct trials can be seen in Table 1. The mean number of errors of commission per subject contributing to the analysis was 20.05 (SD = 9.50; range = 7–57). On average, children were accurate on 68.35% of possible Go and No-go trials and inaccurate on 33.34% of No-Go trials. Children were slower in responding on correct trials (*M* = 552.88 ms, *SD* = 63.79) relative to error trials (M = 455.27, *SD* = 63.31; *t*(1,87) = 20.20, *p* < .01; *d* = 1.54).

**Table 1:** Behavioral performance on error and correct trials in the Go/No-Go task.

	Mean	*SD*	Range
Error trials	20.05	*(9.50)*	7–57
Correct trials	168.20	*(37.98)*	50–235
Percent error on No-Go trials	33.34%	*(0.15)*	8.75–81.94%
Percent correct	68.35%	*(0.10)*	23.13–75.00%
Reaction time error	455.27	*(63.31)*	337.49–650.85
Reaction time correct	552.88	*(63.79)*	402.04–717.60

### 3.2 ERP measures

#### 3.2.1 The ERN and CRN

**Fig. 3:**
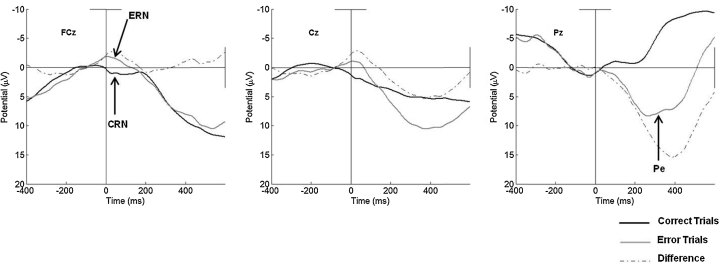
Response-locked error, correct, and difference waveforms for the total sample at FCz, Cz, and Pz.

The response-locked waveforms at electrode sites along the midline at FCz, Cz, and Pz can be seen in Fig. 3. Visual inspection of the waveforms reveals a negative deflection around the time of error commission relative to correct responses at both frontal sites along the midline, FCz and Cz. Average amplitudes on error and correct trials, as well as the difference between them, can be seen in Table 2. The presence of an ERN was further assessed with a three (Site: FCz, Cz, and Pz) × two (Trial Accuracy) ANOVA. As is illustrated in Fig. 3, main effects revealed greater negativity on error trails (*F*(2,87) = 18.44, *p* < .001, partial *η*^2^ = .18), as well as at the fronto-central sites (*F*(2, 174) = 15.70, *p* < .001, partial *η*^2^ = .29). Further confirming the presence of the ERN, in addition, was a significant interaction between Site and Trial Accuracy, (*F*(2, 174) = 29.83, *p* < .001, partial *η*^2^ = .38) revealing differences between error and correct trials as a function of electrode site, with greater negativity at frontal relative to posterior sites on error trials.

**Table 2:** Mean (SD) ERN, CRN, and Pe amplitudes at FCz, CZ, CPz, and Pz.

Components		Midline electrode sites		
	FCz	Cz	CPz	Pz
ERN	−2.79 (5.08)	−1.52 (5.11)	0.64 (1.05)	1.80 (5.79)
CRN	0.54 (3.74)	2.17 (3.83)	3.11 (4.84)	1.60 (4.81)
ΔERN	−3.32 (5.10)	−3.67 (5.49)	−2.42 (5.67)	0.24 (6.72)
Pe	5.92 (8.93)	9.00 (8.58)	10.11 (8.96)	8.04 (10.55)
Pe correct	6.89 (6.49)	5.65 (5.89)	2.11 (6.89)	−4.48 (9.30)
ΔPe	−0.94 (6.74)	3.35 (7.00)	8.00 (7.77)	12.47 (8.96)

Follow-up paired-samples *t*-tests revealed that amplitudes at both FCz (t(87) = 6.06, *p* < .001, *d* = .75) and Cz (t(87) = 6.34, *p* < .001 *d* = .82) were more negative on error relative to correct trials. In contrast, there were no differences between the amplitudes of the ERN and CRN at Pz (*t*(87) = −.28, *p* = .78, *d* = .04). When considering the amplitude of the ERN alone, it was observed to be larger at FCz than at Cz (*t*(87) = 4.09, *p* < .001, *d* = .25), suggesting that the ERN was maximal at FCz. However, the mean difference between the ERN and CRN (ΔERN) was comparable at FCz and Cz (*t*(87) = .9, *p* < .39, *d* = .03).

#### 3.2.2 The Pe

Examination of Fig. 3 also revealed the presence of the Pe at Pz in contrast to frontal sites, located posteriorly along the midline. Confirming this, results from a three (Site: FCz, Cz, and Pz) × two (Trial Accuracy) ANOVA revealed a significant main effect of region (*F*(2,174) = 17.61, *p* < .001, partial *η*^2^ = .29), suggesting that there was greater positivity at posterior sites across both error and correct trials. In addition, a main effect of accuracy was identified (*F*(2,187) = 66.07, *p* < .001, partial *η*^2^ = .43), with greater positivity observed on error relative to correct trials. Finally, a significant interaction between Site and Trial Accuracy was also found, (*F*(2, 174) = 110.34, *p* < .001, partial *η*^2^ = .60) indicating that the differences between error and correct trials varied as a function of electrode site, with greater positivity at posterior relative to frontal sites on error trials.

The mean amplitudes and difference scores at midline locations for the Pe can be found in the bottom two rows of Table 2. Post hoc paired-samples tests indicated that the amplitude of the Pe was significantly more positive on error relative to correct trials at Cz (*t*(87) = −4.49, *p* < .01, *d* = .45) and Pz (*t*(87) = −13.16, *p* < .001, *d* = 1.25). There were no differences in the amplitude of the Pe on error trials at Cz and Pz, however, as can be seen in Fig. 3, the difference between amplitudes on error relative to correct trials (ΔPe) was significantly greater at Pz than at Cz (*t*(87) = 4.09, *p* < .001, *d* = .53).

### 3.3 Links between behavior and ERP measures

Relations between children's error monitoring were first explored by relating the ERN, CRN, and ΔERN at FCz to accuracy and reaction time. Results of these analyses, which can be seen in Table 3, revealed a significant negative correlation between the CRN and the percentage of errors children made within the task (*r* = −.27, *p* = .01) as well as between ΔERN and trial accuracy (*r* = −.23, *p* = .03). No associations between these components and reaction time were observed.

**Table 3:** Correlations between ERP amplitudes and behavioral performance on the zoo task.

	FCz			Pz		
	ERN	CRN	ΔERN	Pe	Pe Correct	ΔPe
Percent error	−.03	−.27*	.16	−.04	.07	−.13
Percent correct	−.11	.16	−.23*	.11	−.20	.34**
Reaction time error	.20	.01	.19	.03	.13	−.11
Reaction time correct	.18	.14	.08	.06	.16	−.09

* *p* < .05.

** *p* < .01.

Similarly, there were limited associations between the Pe at Pz on error trials and children's behavioral performance, with the exception of a positive correlation between task accuracy and the ΔPe (*r* = 34, *p* = .001). This suggests that children who respond more accurately on Go trials in the Go/No-go task exhibit greater differences in Pe amplitude on error relative to correct trials.

### 3.4 Relating child characteristics and behavioral performance

After examining within-task performance, associations between performance and children's age in months and gender were explored through the use of simultaneous regression analyses for individual behavioral outcomes. As can be seen in the regression results reported in the top left portion of Table 4, analyses revealed that with monthly increases in age, children were increasingly accurate on no-go trials. In contrast, no association between age and the commission of errors was identified. Age was also related to children's speed on both error and correct trials, with increasing age associated with increasingly fast performance on trials in which children responded correctly and incorrectly. Thus, the increase in accuracy on no-go trials is not because of a speed-accuracy tradeoff.

**Table 4:** Simultaneous regression results: unstandardized regression coefficients, standard errors, and *R*^2^ values of associations between behavior, ERP components, and children's age and gender.

	Age	Gender	Error Rate	
	*B(SE)*	*B(SE)*	*B(SE)*	*R*^*2*^
*Behavioral data*				
Percent error	−0.19 (.15)	6.65 (3.16)*		.06
Percent correct	0.56 (0.08)**	−0.38 (1.75)		.34
Reaction time error	−2.67 (0.60)**	1.12 (12.39)		.19
Reaction time correct	−3.01 (0.58)**	−11.38 (11.94)		.26
*ERP components*				
ERN	0.01 (0.05)	−0.91 (1.14)	−0.03 (0.04)	.01
CRN	−0.01 (0.04)	0.16 (0.81)	−0.07 (0.03)**	.07
ΔERN	−0.01 (0.05)	−1.02 (1.12)	0.06 (0.04)+	.04
Pe	−0.03 (0.11)	−4.77 (2.31)**	−0.00 (0.08)	.06
Pe Correct	−0.20 (0.09)**	−6.98 (1.85)**	0.08 (0.06)	.22
ΔPe	0.19 (0.08)**	3.46 (1.63)**	−0.03 (0.05)	.13

* *p* < .05.

** *p* < .01.

+ *p* < .15.

As for gender differences in performance on the Zoo Task, as is revealed in Table 4, gender did not account for variability in children's ability to respond to correct trials accurately (*M*_Girls_ = 67.76%, *SD* = 9.67; *M*_Boys_ = 68.88%, *SD* = 10.21). Gender was found to significantly contribute to children's errors of commission, with girls committing fewer errors than boys (*M*_Girls_ = 30.13%, *SD* = 12.81; *M*_Boys_ = 36.27%, *SD* = 16.30). Despite differences in accuracy, gender was not related to variability in children's reaction time on error or correct trials.

#### 3.4.1 The role of age and gender in error processing

**Fig. 4:**
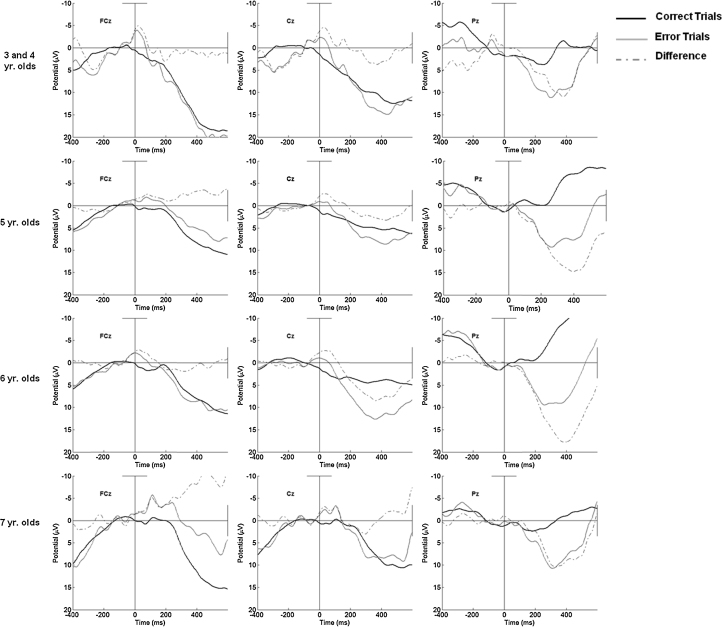
Response-locked error, correct, and difference waveforms for 3–4 (*N* = 11), 5 (*N* = 33), 6 (*N* = 34), and 7 year olds (*N* = 10) at FCz, Cz, and Pz.

In contrast to the relatively strong associations between children's age and behavioral performance on the Zoo Task, as can be seen in the bottom half of Table 4, simultaneous regression analyses, including age in months, gender, and error rates as predictors indicated that neither age nor children's gender accounted for the variation observed in the ERN, CRN, or ΔPe. In contrast, increased error rates did account for variability in CRN amplitude. The response-locked waveforms for groups of children as a function of age are portrayed in Fig. 4. Indeed, as can be seen in the first column of Fig. 4 on the left side, it is clear that an ERN is present at FCz across all age groups, including those 3 and 4 years of age.

**Fig. 5:**
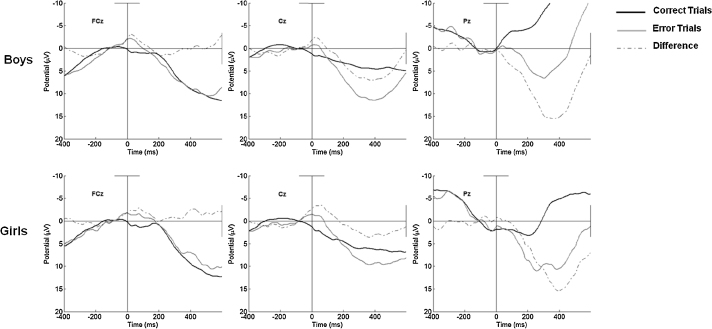
Response-locked error, correct, and difference waveforms as a function of gender at FCz, Cz, and Pz.

Further analyses of the Pe, revealed that children's gender accounted for variance in the amplitude of the Pe at Pz. Specifically, as is portrayed in the third panel of Fig. 5, male gender was significantly associated with decreased amplitude of the Pe on error and correct trials. Analyses involving the ΔPe revealed that gender and increases in age accounted for some of the variance in the difference in amplitude of the Pe on error relative to correct trials. This is to say that as children got older, although Pe amplitude did not change on error trials, the difference between amplitude on error relative to correct trials increased at frontal sites.

## 4 Discussion

In this investigation, behavioral and neurophysiological correlates of young children's cognitive control and error monitoring were explored using a child-friendly, developmentally appropriate Go/No-Go task administered in a school setting to a representative sample of children. The results described above contribute to the growing understanding of developmental changes in ERP components related to error processing. In addition to providing novel information regarding these phenomena in young children, these results provide insight into the complexities of considering developmental changes in the ERN and Pe, revealing the importance of considering additional child-level factors such as gender.

### 4.1 Developmental changes in error monitoring?

What might explain the age-related patterns observed in this investigation? Given that the developmental literature – based largely on behavioral data – provides extensive evidence for rapid growth in the development of executive skills in early childhood, one might expect to observe similar changes in children's error processing during this same period of time. However, whether a particular ERP component will change during that period depends on the specific component of EF that it reflects. It is not surprising that the ERN and Pe would be dissociated during this period, as a great deal of evidence suggests that the ERN and Pe reflect different computations (see, e.g., Gehring et al., 2012; Niuewenhuis et al., 2001; Overbeek et al., 2005)

As can be seen in Fig. 4, our data provide evidence for the presence of the ERN, CRN, and Pe in children as early as 3 years of age but little developmental change in the ERN or CRN during the 3–7 age range. To our knowledge these results reflect the youngest reported ERN, CRN, and Pe on a Go/No-Go task, with waveforms showing the presence of each component even in youngest age groups observed. Previous reports have provided evidence for developmental changes in the ERN from childhood to adulthood (e.g., Davies et al., 2004), The ERN and CRN amplitudes observed in these data were small relative to what is typically seen in adults, but little evidence for developmental differences in the ERN were identified between ages 3 and 7 in this sample. Further complicating questions of development, it is known that the ERN is larger in accuracy emphasis than in speed emphasis conditions (Gehring et al., 1993). To account for this potential issue in our analyses, we added accuracy as a predictor in regressions involving the ERN measures. These analyses failed to detect age-related differences in the ERN. Therefore, taken together with previous studies, the evidence here suggests that the ERN is apparent in children by the age of 4 but undergoes its maximal development during early adolescence.

In contrast, although there have been previous reports including children as young as 7, previous investigations demonstrating a Pe in adolescents, and young adults have indicated that the Pe does not increase with age (Davies et al., 2004, Wiersema et al., 2007). Clear evidence emerged for age-related increases in the Pe in this study. Such a developmental trend fits with the processes thought to be reflected by the Pe and with behavioral literature on the development of the corresponding components of EF. Specifically, if one assumes that the Pe is related to reflective processes involved in error monitoring, it follows that as children develop these relatively complex skills from the ages of 3–7, changes in the Pe might be observed.

### 4.2 Assessing error processing in early childhood

Although there important implications of EF for children's academic and regulatory skills in the classroom setting, it can be quite challenging to assess these abilities across early childhood (Griffin et al., 2014). Given that contextual factors, such as growing up in poverty, are associated with reduced EF ability (Raver et al., 2013), it is particularly important that we understand the development of these skills in children from diverse backgrounds. To overcome some of these challenges associated with obtaining EF data from young children, and to explore the development of these skills in representative groups of children, we feel that there are two important considerations for testing EF. One is to ensure that children are engaged throughout the ERP testing session and are motivated to participate. The second is that barriers to participation should not prevent some children from participating. To this end, all aspects of this investigation were designed to be child-centered and inclusive. In an effort to recruit a more representative sample of children, testing was conducted directly in the school setting. Before beginning the investigation, experimenters visited children's classrooms to provide a brief demonstration describing ERP research methods, explain the project, and answer children's questions. As a result, all children were informed about the process in advance of participation and were enthusiastic about being involved. In contrast to laboratory based experiments, working with children in a space that was familiar also resulted in increased comfort and compliance during assessments.

Building on a measure developed by McDermott and colleagues, we refined the Go/No-go with the goal of making it equally interesting and understandable for children from preschool through early elementary school. To ensure that children had knowledge of the goals of the task and retained engagement throughout, we employed an interesting storyline that even the youngest participants could follow. The children enjoyed seeing the different animal images, and stimuli for go and no-go trials were clear and easy to differentiate. Experimenters involved with data collection had extensive experience working with young children, and pilot testing was conducted to develop assessment strategies specific to young children. Appealing prizes, including books and zoo-themed pencils and stickers were awarded upon completion of the testing session. Based on these experiences, to reduce fatigue and minimize movement artifacts in the data, assessments were fast-paced, with a number of breaks built in for children to move around in between blocks of the task. Attrition is often an issue when conducting ERP experiments with young children. In this investigation, all children were able to complete the task, and only 7 of the 95 children yielded unusable data due to an insufficient number of trials.

It is possible that the efforts made to create a child-friendly testing environment and task also contributed to the age-related patterns in the ERN and Pe reported here. When reviewing the ERN literature involving children, the trend in which more recent studies show the ERN appearing at earlier ages appears to be related to an increase in developmentally sensitive tasks. These results provide further examples of the need for developmentally appropriate measures, as previously discussed by other researchers (e.g., McDermott et al., 2007, Torpey et al., 2012). In a similar way, it is possible that our efforts to create a child-friendly assessment and testing environment directly impacted the quality of the ERP data collected and, in turn, allowed for the observation of error-related components earlier in childhood than previously reported. For example, McDermott and colleagues (2007) found differences in young children's behavioral performance as a function of stimulus differences on a Flanker task. If the ERN represents some sort of fundamental mechanism of cognitive control that is present in young children, creating measures with stimuli that are engaging and familiar may provide the potential to elicit the ERN at different ages. Indeed, to the extent that the age-related trends in the ERN presented here diverge from previous reports, it seems increasing likely that issues related to the testing and measurement of error processing appear to be particularly important for assessing the youngest groups of children.

### 4.3 Considering behavior and ERP components in concert

Behavioral data from the Go/No-go task revealed that children's performance became increasingly efficient and accurate with age. However, little evidence for relations between behavioral performance and related ERPs was identified. Moreover, as discussed above, despite clear developmental trends observed in behavioral data, age-related changes were not seen in the ERN within the same task. If it is the case that children's EF abilities – as assessed using behavioral measures including accuracy and reaction time on measures of response inhibition such as the go/no-go – are important for their academic skills, it follows that it would be of interest to investigate the ERP components elicited by these same tasks. However, these data demonstrate that, even when controlling for age, the relations between children's behavioral performance and their error monitoring (as reflected by the ERN, CRN, and Pe) appear to be minimal. Our findings suggest that these different forms of data reflect different aspects of executive skills; the different measures should not necessarily be seen as interchangeable when used to evaluate these processes in young children.

### 4.4 The role of gender

When considering children's performance as a function of gender, an interesting pattern of association was revealed. In terms of children's accuracy and reaction time assessed behaviorally, although children became increasingly able to correctly respond to Go trials with age, girls were far better at resisting error commission than boys, regardless of age. These differences in the commission of errors did not translate to differences in the components associated with initial error processing; however, the discrepancy between girls later processing of errors relative to correct trails was accompanied by corresponding gender differences in the Pe on error and correct trials and the ΔPe. Given that gender differences are not consistently seen in children and adults, more work is needed to understand the role of gender in error processing.

One potential source of selection bias suggests that future work on gender differences would benefit from a more systematic randomized selection procedure. Our subject recruitment involved an engaging presentation to parents that included demonstrations and a discussion of the importance of EF. In addition, there was substantial word-of-mouth interest generated among parents. Although equal numbers of boys and girls participated in the investigation, it is possible that parents of boys might be more interested in enrolling their children in the investigation because of perceived EF deficits in male children relative to females, potentially contributing to gender differences observed here. Further study is needed to establish whether a population-level gender difference among boys and girls truly lies at the heart of the findings observed in this sample.

### 4.5 Limitations and future directions

Data from this investigation provide ERP evidence for error processing in children as young as 3 years old. However, considering the results reported here in the context of the growing body of evidence related to the ERN and Pe across development, it is clear that a great deal of additional work must be done to explore these processes as they are developing. Longitudinal data across important transitional periods in childhood through adolescence would provide much-needed information regarding both the developmental trajectories and individual variability in these components over time. Moreover, given the evidence for developmentally-appropriate measures, it would also be helpful to explore the range of child-level factors – including task engagement and motivation – that might contribute to children's performance. Richer measures of task engagement (such as measures of facial expression and affect) might assist in that effort.

## Conflict of interest

The authors declare no conflict of interest.
